# Chaperone-Mediated Autophagy Targets IFNAR1 for Lysosomal Degradation in Free Fatty Acid Treated HCV Cell Culture

**DOI:** 10.1371/journal.pone.0125962

**Published:** 2015-05-11

**Authors:** Ramazan Kurt, Partha K. Chandra, Fatma Aboulnasr, Rajesh Panigrahi, Pauline Ferraris, Yucel Aydin, Krzysztof Reiss, Tong Wu, Luis A. Balart, Srikanta Dash

**Affiliations:** 1 Department of Medicine, Division of Gastroenterology and Hepatology, Tulane University School of Medicine, New Orleans, Louisiana, United States of America; 2 Pathology and Laboratory Medicine, Tulane University School of Medicine, New Orleans, Louisiana, United States of America; 3 Neurological Cancer Research, Stanley S Scott Cancer Center, LSU Health Science Center, New Orleans, Louisiana, United States of America; University College London, UNITED KINGDOM

## Abstract

**Background:**

Hepatic steatosis is a risk factor for both liver disease progression and an impaired response to interferon alpha (IFN-α)-based combination therapy in chronic hepatitis C virus (HCV) infection. Previously, we reported that free fatty acid (FFA)-treated HCV cell culture induces hepatocellular steatosis and impairs the expression of interferon alpha receptor-1 (IFNAR1), which is why the antiviral activity of IFN-α against HCV is impaired.

**Aim:**

To investigate the molecular mechanism by which IFNAR1 expression is impaired in HCV cell culture with or without free fatty acid-treatment.

**Method:**

HCV-infected Huh 7.5 cells were cultured with or without a mixture of saturated (palmitate) and unsaturated (oleate) long-chain free fatty acids (FFA). Intracytoplasmic fat accumulation in HCV-infected culture was visualized by oil red staining. Clearance of HCV in FFA cell culture treated with type I IFN (IFN-α) and Type III IFN (IFN-λ) was determined by Renilla luciferase activity, and the expression of HCV core was determined by immunostaining. Activation of Jak-Stat signaling in the FFA-treated HCV culture by IFN-α alone and IFN-λ alone was examined by Western blot analysis and confocal microscopy. Lysosomal degradation of IFNAR1 by chaperone-mediated autophagy (CMA) in the FFA-treated HCV cell culture model was investigated.

**Results:**

FFA treatment induced dose-dependent hepatocellular steatosis and lipid droplet accumulation in HCV-infected Huh-7.5 cells. FFA treatment of infected culture increased HCV replication in a concentration-dependent manner. Intracellular lipid accumulation led to reduced Stat phosphorylation and nuclear translocation, causing an impaired IFN-α antiviral response and HCV clearance. Type III IFN (IFN-λ), which binds to a separate receptor, induces Stat phosphorylation, and nuclear translocation as well as antiviral clearance in FFA-treated HCV cell culture. We show here that the HCV-induced autophagy response is increased in FFA-treated cell culture. Pharmacological inhibitors of lysosomal degradation, such as ammonium chloride and bafilomycin, prevented IFNAR1 degradation in FFA-treated HCV cell culture. Activators of chaperone-mediated autophagy, including 6-aminonicotinamide and nutrient starvation, decreased IFNAR1 levels in Huh-7.5 cells. Co-immunoprecipitation, colocalization and siRNA knockdown experiments revealed that IFNAR1 but not IFNLR1 interacts with HSC70 and LAMP2A, which are core components of chaperone-mediated autophagy (CMA).

**Conclusion:**

Our study presents evidence indicating that chaperone-mediated autophagy targets IFNAR1 degradation in the lysosome in FFA-treated HCV cell culture. These results provide a mechanism for why HCV induced autophagy response selectively degrades type I but not the type III IFNAR1.

## Introduction

Hepatitis C virus (HCV) infection can lead to chronic liver disease, liver cirrhosis and hepatocellular carcinoma, making it a major public health problem worldwide [1–3]. The new standard of care for chronic HCV genotype 1 infection includes a combination of interferon-alpha (IFN-α ribavirin, and one protease inhibitor or polymerase inhibitor [4]. This combination therapy has greatly improved the sustained antiviral response among chronic HCV patients. Unfortunately, it has not had the same effect on the sustained virological response among patients who are non-responders to pegylated IFN-α and ribavirin [5,6]. The poorly sustained virological response to this new triple therapy among patients who are non-responders to the IFN-α and ribavirin is a major challenge in treating chronic HCV infection. The host and viral related factors that have been implicated in the poor response to antiviral therapy include IL-28B genotypes, viral load, viral genotypes, body weight, stage of the liver disease, type-2 diabetes mellitus (DM), fibrosis stage, race, and co-infection with human immunodeficiency virus (HIV) [7,8]. We propose that a better understanding of the mechanisms of HCV clearance by type I, type II and type III IFN using relevant cell culture may lead to novel strategies to improve treatment response and reduce the burden of liver cirrhosis and cancer.

Hepatic steatosis is defined as the accumulation of lipid droplets in hepatocytes, which can be either microvesicular or macrovesicular steatosis depending on the size of the lipid droplets [9,10]. Hepatitis C virus infection has been implicated to induce hepatic lipogenesis and pathological features of hepatic steatosis [11,12]. Recent studies indicate that structural and nonstructural proteins of HCV activate various pathways of lipid metabolism and lipogenic gene expression, resulting in increased accumulation of lipid droplets in infected hepatocytes [13,14]. The presence of hepatic steatosis in patients with chronic HCV infection is much higher than in the uninfected population [15,16]. Additionally, the increased prevalence of fatty liver in chronic hepatitis C patients is also associated with excessive alcohol consumption, increased body weight, DM, and other metabolic diseases [16]. A number of clinical studies have reported that obesity is becoming one of the highest risk factors for non-responsiveness to HCV therapy [7–22]. The mechanism by which intracellular lipid accumulation in the liver reduces the sustained virological response to HCV therapy is unknown.

The most abundant free fatty acids (FFAs) in the liver triglycerides in patients with nonalcoholic fatty liver disease are palmitic and oleic acids [23]. We have shown that long-term co-culturing of FFA and HCV in a cell culture model induces endoplasmic reticulum (ER) stress-mediated autophagy response and downregulates IFNAR1, leading to the creation of defective Jak-Stat signaling and impaired antiviral response of IFN-α [24]. However, the FFA-induced cellular stress and autophagy response has no effect on the expression of interferon-lambda receptors. We report here that chaperone-mediated autophagy (CMA) selectively targets IFNAR1 degradation in the lysosome in FFA-treated HCV cell culture, whereas IFN-λ (Type III IFN) may induce Stat-phosphorylation and nuclear translocation, thus clearing HCV replication in free fatty acid cell culture that is resistant to IFN-α.

## Materials and Methods

### Cell culture and chemicals

The Huh-7.5 human hepatocellular carcinoma cell line was obtained from the laboratory of Dr. Charles M. Rice (Rockefeller University, New York). Huh-7 and Huh-7.5 cells lines were maintained in Dulbecco’s Modified Eagle Medium (DMEM; Life Technologies, Carlsbad, CA), supplemented with 2 mM L-glutamine, sodium pyruvate, nonessential amino acids, 100U/mL penicillin, 100mg/mL streptomycin and 10% fetal bovine serum (FBS). The Renilla luciferase reporter based pJFH1-ΔV3-Rluc clone used in our experiment has been described previously [24]. The following reagents were purchased from commercial sources: IFN-α (EMD Merck, Billerica, MA); IFN-λ1 and IFN-λ2 (PeproTech, Rocky Hill, NJ); Oil red O, sodium oleate, sodium palmitate, fatty acid free bovine serum albumin (BSA), 6-Aminonicotinamide (6-AN) (Sigma-Aldrich, St. Louis, MO); Synthetic siRNA duplexes targeted to STAT1, STAT2, STAT3 and LAMP2A (Qiagen, Maryland, USA); antibodies specific for IFNαR1 (Santa Cruz Biotechnology, CA), IFNαR2, IFNγR1, (Santa Cruz Biotechnology, Santa Cruz, CA), IFN-λ receptor IL10Rβ (R&D Systems, Minneapolis, MN), antibodies to HCV Core protein (Thermo Fisher Scientific, Waltham, MA), antibodies to Stat1, Stat2 and Stat3 (Cell Signaling, MA).

### Free Fatty Acid-treated HCV cell culture

Huh-7.5 cells cultured in 100-mm petri dish were infected with cell culture grown HCV (JFH-∆V3-Rluc) with a multiplicity of infection (MOI) of 0.1. The following day the infected Huh-7.5 culture was washed with PBS then incubated with 10 mL of DMEM and 10% FBS [25,26]. Infected Huh-7.5 cells were cultured long-term by splitting at a 1:10 ratio at 6-day intervals. Replication of HCV in the infected cell culture at each interval was confirmed by measuring the Renilla luciferase activity and core protein expression by immunostaining and Western blotting [26]. We prepared a mixture of oleate and palmitate at a ratio of 2:1 that mimics benign chronic hepatic steatosis due to free fatty acids (FFA). Briefly, 100 mM oleate (Sigma Catalog No. O-7501) and 100 mM palmitate (Sigma catalog No. P-0500) stocks were prepared in 0.1 M NaOH at 70°C using a water bath and filter sterilized. Five percent Bovine Serum Albumin (BSA) was prepared in distilled water and filter sterilized. We prepared a 5mM working stock solution for oleate and palmitate separately in 5% BSA solution in distilled water at 60°C, then stored the mixture at room temperature. Oleate and palmitate were first mixed together at a 2:1 ratio in a sterile environment under laminar flow each time prior to use. FFA mixture was added to HCV-infected Huh-7.5 cells to a final concentration of 100 μM. Previous studies from our laboratory and others determined that FFA used in the concentration range of 10–100 mM has minimum hepatocellular toxicity in HCV-infected cell culture [24,27].

### Oil Red O staining

Lipid droplet accumulation in HCV culture was visualized by Oil Red O (Sigma-Aldrich, Cat log No. O0625 St Louis, MO) staining as described previously [28]. Briefly, persistently HCV-infected Huh-7.5 cells cultured with or without FFA were harvested then fixed in 10% formalin in PBS for one hour, then stained with Oil Red O solution for one hour. The following methods were used to prepare Oil O Red solution. A stock solution was prepared by mixing 0.5 gm of Oil Red O powder in 100 ml of isopropanol. The solution was filtered through double layer Whatman filter paper. A freshly prepared 10 ml of Oil Red O solution was prepared by adding 6ml of stock with 4ml of double distilled water and incubated with FFA treated culture. After this treatment, the cells were washed with PBS and counterstained with either haematoxylin for light microscopy evaluation or Hoechst dye (H33342, Calbiochem, Darmstadt, Germany) (10 μg/mL prepared in PBS) for confocal microscopy.

### Immunostaining for HCV Core

Infected Huh-7.5 cells with or without IFN treatment were mounted onto a glass slide using the cytospin method. The cells were washed twice with 10 mmol/L PBS (pH 7.4) for 5 minutes. After this step, cells were fixed in chilled acetone for 15 minutes and then permeabilized by treatment with Reveal Decloaker RTU reagent (RV 100; Biocare Medical, Concord, CA) for 25 minutes at its boiling point. Then, slides were cooled to room temperature for 25 minutes. Slides were treated with a background-sniper blocking reagent (BS966; Biocare Medical) for 10 minutes at room temperature. The cells were incubated with monoclonal anti-core antibody (MA1-080; Thermo Fisher Scientific, Rockford, IL) and diluted 1:200 with Da Vinci Green diluent (PD900; Biocare Medical) for 1 hour at room temperature. After the primary antibody incubation, the cells were washed three times in Tris-buffered saline (pH 8.0), and incubated with MACH 4 mouse probe (UP534; Biocare Medical) for 10 minutes. The cells were then incubated with MACH 4 horseradish peroxidase polymer (MRH534; Biocare Medical) for 30 minutes, and washed with Tris-buffered saline three times. Next, the cells were treated with diaminobenzidine chromogen (Dako, Carpinteria, CA) for 5 minutes. The slides were counterstained with hematoxylin for 30 seconds and Tacha’s bluing solution (HTBLU; Biocare Medical) for 30 seconds and then were dehydrated, mounted, and observed via light microscopy.

### Western blotting

Western blotting was performed using a standard protocol established in our laboratory. Infected Huh-7.5 cells were washed twice with PBS and then lysed in ice-cold RIPA buffer. Total protein content of the extract was quantified using a Bio-Rad protein assay kit (Bio-Rad, Hercules, CA). Equal amounts of proteins were loaded on SDS-PAGE gels and Western blotting was carried out using antibodies to STAT1, p-STAT1, STAT2, p-STAT2, HSC70, β-actin and GAPDH (Cell Signaling Danvers, MA); as well as LAMP2A (Abcam).

### Confocal microscopy

Cell surface expression of IFN-α, IFN-γ, and IFN-λ receptors (IFNAR1, IFNγR1, and IL10Rβ, respectively) at Huh7 cells with and without FFA were examined at 4°C using a published protocol [29]. Briefly, 1 X 10^6^ Huh-7 cells in ice-cold DMEM containing 1% (v/v) FBS were incubated for 1 hour at 4°C with primary antibody (1:25 dilution) specific for IFN-α or IFN-γ, or IFN-λ receptors with gentle shaking. Cells were then washed once with DMEM containing 1% (v/v) FBS and then incubated with Alexa Fluor 488 (green) conjugated secondary antibody (dilution of 1:50) (Life Technologies) for 30 minutes at 4°C with shaking. Finally, cells were washed twice with DMEM containing 1% (v/v) FBS and fixed with 4% paraformaldehyde. Cells were covered with a few drops of Oil Red O stain for 30 minutes. Cell suspension was then examined for IFN-receptor expression via confocal microscopy.

### Nuclear Translocation Assay

Uninfected and HCV infected Huh-7.5 cells were seeded in two-well chamber slides (Thermo Fisher Scientific) at a density of 2 X 10^4^ cells per well. After overnight incubation, cells were transfected with 500 ng of pSTAT1-GFP or pSTAT2-GFP plasmid using FuGENE 6 (Roche Diagnostics, Indianapolis, IN) transfection reagent. At 48 hours, culture was treated with either IFN-α or IFN-λ (2.5 x IC_90)_ for one hour and then fixed with 4% paraformaldehyde. HCV core staining was performed using a monoclonal antibody at a dilution of 1:200 (MA1-080; Thermo Fisher Scientific, Rockford, IL) and secondary Alexa 594 labeled goat anti-mouse antibody at a dilution of 1:500 (Life Technologies, Carlsbad, CA). After the staining Hoechst 33342 nuclear dye (Calbiochem, Darmstadt, Germany) was added to the samples at 1 μg/ml, and incubated for five minutes in PBS. The translocation of GFP as well as the HCV core was monitored using a Leica TCS SP2 confocal microscope equipped with three lasers (Leica Microsystems, Exton, PA). Optical slices were collected at 512 × 512 pixel resolution.

### Co-immunoprecipitation

The interaction of LAMP2A and HSC70 with IFNAR1 was examined by co-immunoprecipitation using a protocol described previously with slight modifications [30]. Huh-7.5 cells were cultured at 90% confluence in a 100mm tissue culture dish. After 12 hours of serum starvation, cells were washed once with 10ml of PBS and harvested in 3ml of ice-cold RIPA buffer (0.15 mM NaCl, 0.05mM tris-HCl, pH.7.5, 1% triton, 0.1% SDS, 0.1% NP-40 and 1X protease and phosphatase inhibitor cocktail) on a shaker for 30 minutes. One mg of total extract was immunoprecipitated with antibodies (IFNAR1 1:1000 or IFNLR1:1000) overnight at 4°C on a shaker. Approximately 20μl of protein A/G plus-agarose (Santa Cruz Biotechnology) was added at 4°C for one hour. After this step the beads were washed three times with washing buffer (RIPA buffer). The pellet was suspended in 8ul of SDS-PAGE loading buffer and the samples were boiled for 5 minutes. The supernatant was collected and 20μl of lysate were loaded onto 12% acrylamide gene electrophoresis and Western blotting was performed using antibodies to LAMP2A (Abcam), Interferon-alpha receptor-1 (IFNAR1) (Santa Cruz), Interferon-lambda receptor-1 (IFNLR1) (Sigma) and HSC70 (Cell Signaling) as described before.

### SiRNA transfection

siRNA oligonucleotides specific for Stat1, Stat2, Stat3 and LAMP2A were synthesized by Invitrogen. All-star negative siRNA (Qiagen) was used as a negative control. Sequences of the siRNAs targeting the indicated proteins are as follows: siLAMP2A 5’-CUGCAAUCUGAUUGAUUAUU-3’, siStat1 5’-CGAGAGCUGUC UAGGUUAATT-3’, siStat2 5’-GGCUCAUUGUGGUCUCUAATT-3’, siStat3 5’-CGUU AUAUAGGAACCGUAATT-3’ and scrambled siRNA. Huh-7.5 cells were seeded in a 6-well plate (1 × 10^5^ cells per well). After 24 hours, cells were transfected with 25pmol and 50pmol of siRNA per well using Lipofectamine 2000 (Invitrogen) according to the manufacturer’s instructions.

### Statistical Analysis

All measurements were made at least in triplicate. All results were expressed as mean ± SD (standard deviation). Comparison between two groups was performed with a Student’s t-test. To compare means within groups we performed one factor analysis of variance (ANOVA) using the GraphPad Prism software. We assumed that all measurements have normal probability distributions, which is expected for these types of data. p-value for the ANOVA analysis was significant when p<0.05.

## Results

### FFA induces macrovesicular steatosis and increases HCV replication in Huh-7.5 cells

**Fig 1:**
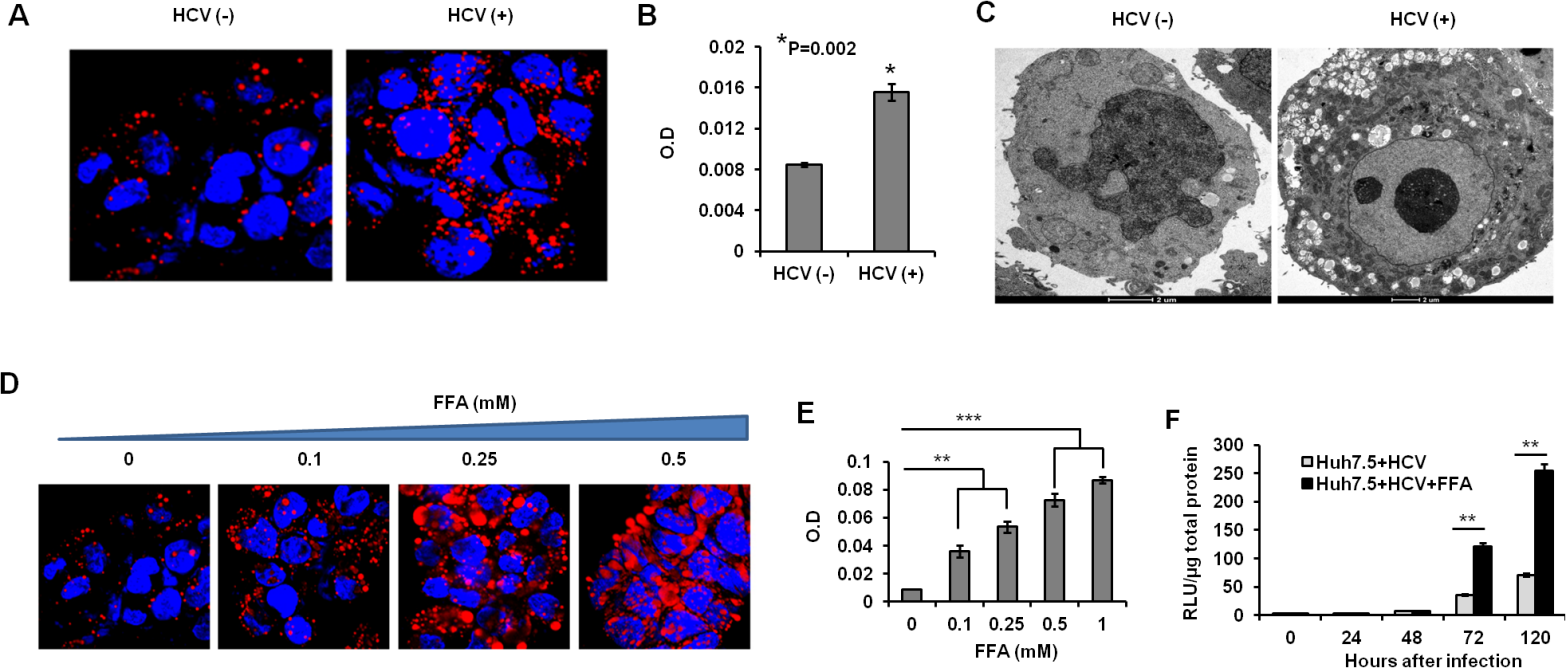
HCV replication induces microvesicular steatosis in persistently infected Huh-7.5. (**A).** Confocal microscopy showing increased accumulation of lipid droplets in Huh-7.5 cells with or without HCV infection. Red: Oil-red staining and green: HCV core expression. The images were taken at 40X magnification. (**B).** Microfluorometer analysis of intracellular fat accumulation in Huh-7 cells with or without HCV replication. The OD values are compared between uninfected and HCV infected cells. (**C).** Electron micrograph showing more intracellular lipid droplet accumulation in persistently HCV infected as compared to uninfected Huh-7.5 cell. (**D).** FFA treatment induces macrovesicular steatosis in Huh-7.5 cells. Uninfected Huh-7.5 cells were treated with increasing concentration of Oleate/Palmitate (2:1 ratio) for 24 hours and hepatocellular steatosis was examined by confocal microscopy after Oil-red staining. (**E**). Microfluorometer analysis shows dose-dependent intracellular fat accumulation in Huh-7.5 cells treated with oleate and palmitate (FFA). OD values are compared between untreated and treated cells. **F.** FFA treatment support HCV replication in cell culture. Huh-7.5 cells were infected with JFH1-ΔV3-Rluc and then treated with FFA 0.5mM. Replication of HCV was measured by Renilla luciferase activity.

A number of reports have claimed that HCV infection alters lipogenesis and directly induces hepatocellular steatosis, therefore the degree of hepatocellular steatosis in the HCV-infected Huh-7.5 cells was quantitatively measured after Oil Red O staining. Using confocal microscopy, we observed an increased number of visible small lipid droplets called microvesicular steatosis in the cytoplasm of HCV-infected Huh-7.5 cell culture as compared to uninfected Huh-7.5 cells (**Fig 1A**). Quantitative assessment of intracellular fat content, performed by microfluorometry, indicated that HCV replication in Huh-7.5 cells induces significantly higher lipid droplet accumulation (**Fig 1B**). Electron microscopic examination of HCV-infected Huh-7.5 cells verified the accumulation of small lipid droplets in the cytoplasm as compared to uninfected cells (**Fig 1C**). Clinical studies have demonstrated that steatosis associated with viral factors (viral steatosis) is not a negative predictor of sustained virological response to IFN-α and RBV. However, chronic hepatitis C with high-grade (grade 2 or 3) macrovesicular steatosis (that is associated with non-alcoholic fatty liver disease or obesity) has been found to be a negative predictor of response to IFN-based antiviral therapy [17]. To understand the molecular basis of intracellular lipid accumulation on IFN-α antiviral response, we developed a co-culture model of fatty acid and HCV in Huh-7.5 cells. Oleate (unsaturated) and palmitate (saturated) fatty acids were mixed together and directly added to the HCV-infected Huh-7.5 cells to induce macrovesicular steatosis. FFA at a concentration of 0.1mM to 0.5mM has no effect on the viability of Huh-7.5 cells and this was demonstrated in our previous publication [24]. The FFA concentration used in this cell culture model is comparable to the pathological range of triglycerides in a human with non-alcoholic fatty liver disease [23]. Co-culture of FFA with HCV infected Huh-7.5 cells showed dose-dependent accumulation of larger size fat vacuoles called macrovesicular steatosis at 24 hours as seen in non-alcoholic fatty liver disease (**Fig 1D**). Quantitative assessment of fat content determined by microfluorometry showed a dose-dependent increase in hepatocellular steatosis in the FFA-treated HCV-infected Huh-7.5 cells (**Fig 1E**). FFA treatment of HCV culture increased viral replication, as confirmed by increased NS5A-Renila luciferase activity (**Fig 1F**). These results suggest that co-culture of free fatty acids with HCV induces hepatocellular steatosis and supports HCV replication.

### Antiviral potency of Type I and Type III IFN against HCV

**Fig 2:**
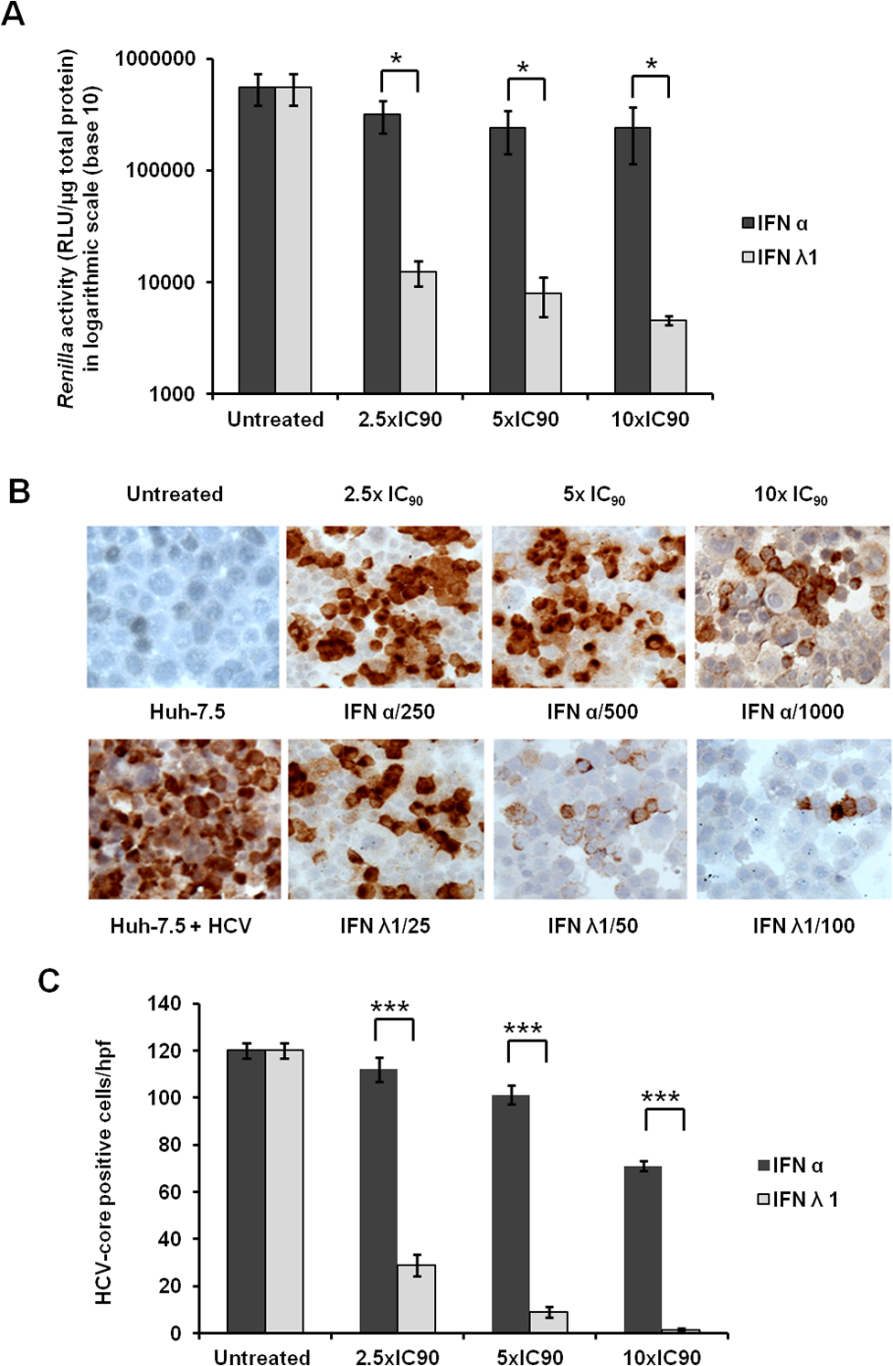
Persistently infected Huh-7.5 cells were treated with 1xIC_90_, 2.5 × IC_90_, and 5 × IC_90_ IFN-α or IFN-λ. After 72 hours antiviral efficacy of IFN-α and IFN-λ was determined by the measurement of *Renilla* luciferase activity and immunohistochemistry for HCV Core protein expression. **(A).** Normalized *Renilla* luciferase activity of culture treated with equivalent concentrations of IFN-alpha or IFN-λ. **(B).** Representative picture showing the expression of HCV core protein in the IFN-treated cells and control uninfected Huh-7.5 by immunostaining. **(C).** Quantification of HCV Core^+^ cells in 10 different high-power fields (×40), compared with untreated control. Both assays confirmed that the antiviral effect of IFN-λ is significantly stronger than IFN-α when used at equivalent concentrations (* p< 0.03, *** p< 0.001).

**Fig 3:**
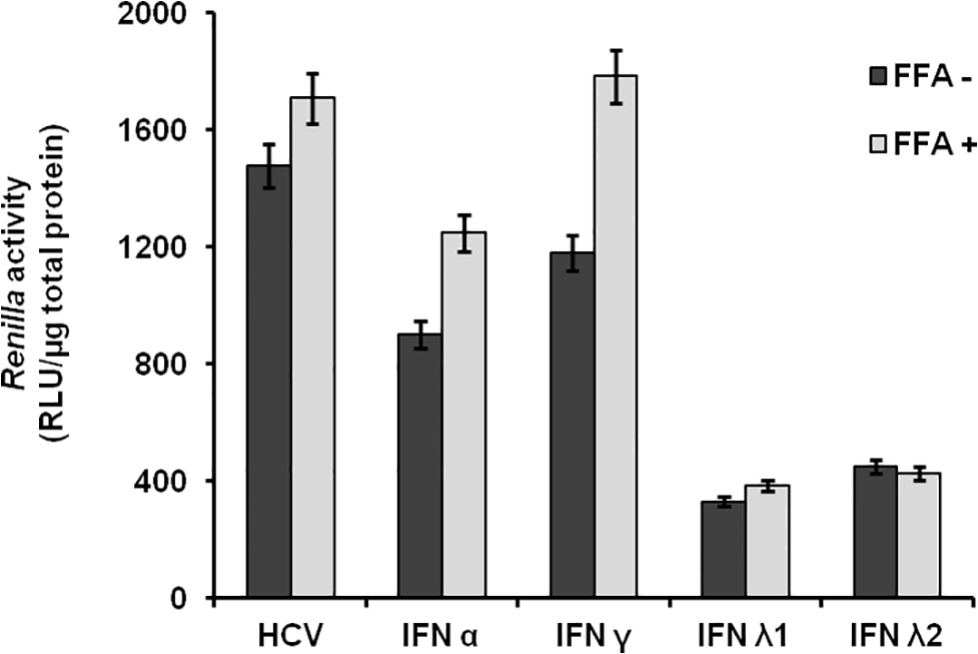
FFA treatment blocks the antiviral response of IFN-α and IFN-γ against HCV. Infected Huh-7.5 cells were cultured in the presence of FFA for 72 hours and then treated with IFN for additional 72 hours. The relative antiviral effect of IFN-α, IFN-γand IFN-λ was compared in the presence and absence of FFA by measuring *Renilla* luciferase activity. The luciferase values are normalized with total protein.

**Fig 4:**
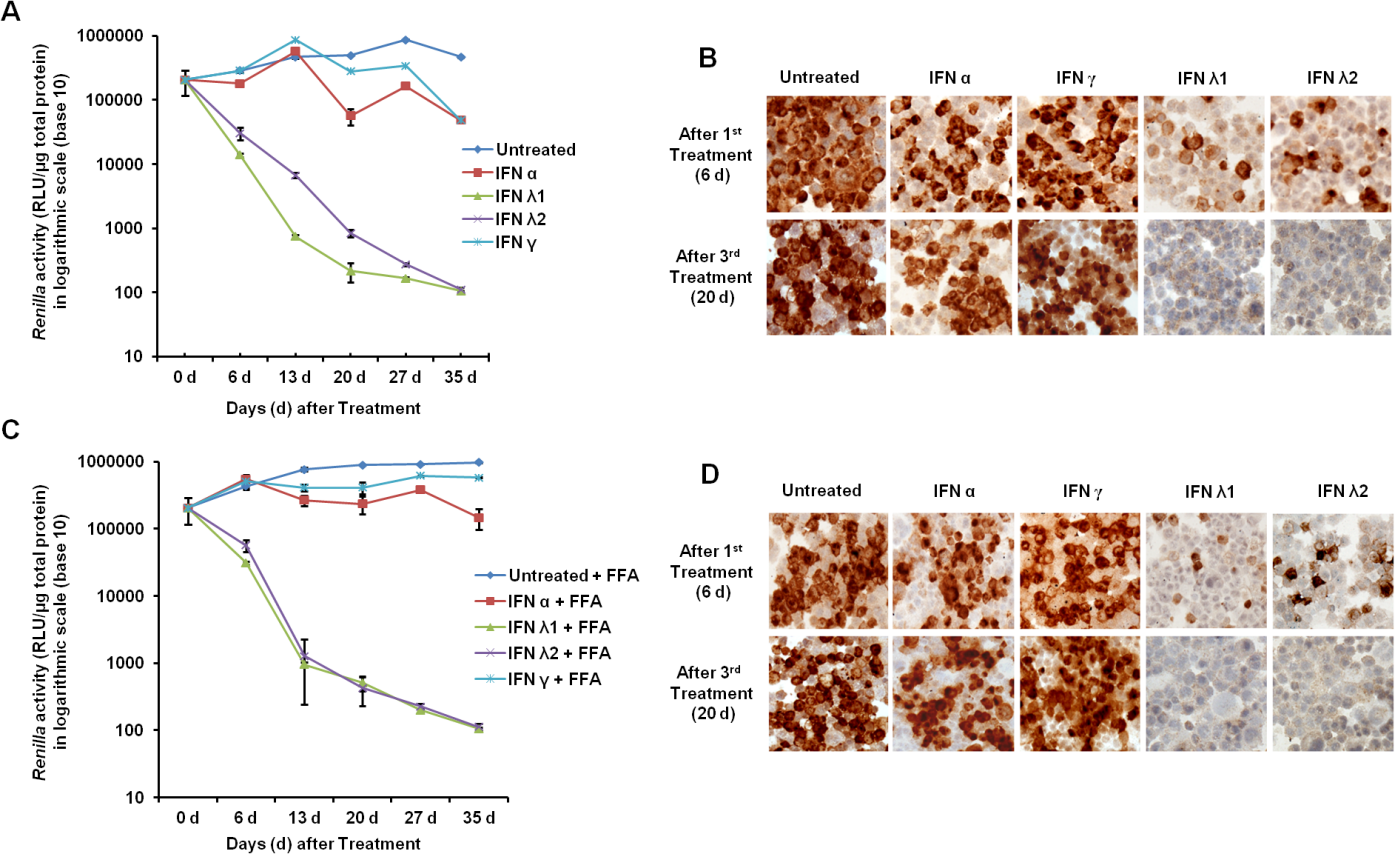
Long-term antiviral response of IFN-α, IFN-γ, and IFN-λ against HCV culture with or without FFA treatment. HCV infected culture was continuously treated with each IFN and antiviral activity was determined by measuring *Renilla* luciferase activity of cell lysates. (**A**)**.** Represents normalized *Renilla* luciferase values of HCV infected culture with or without interferon treatment. (**B**)**.** Shows the expression of HCV core protein in the IFN-α, IFN-γ, and IFN-λ treated cultures at day 6 and day 20. (**C).** Represents normalized luciferase values of FFA treated HCV infected culture with or without interferon. (**D).** Expression of core protein in the in the FFA treated HCV culture after IFN-α, IFN-γ, and IFN-λ treatment at day 6 and day 20.

Previously, we published that persistent HCV infection selectively blocks type I IFN signaling but not type III IFN signaling [25]. In this study we tested whether type III IFN could overcome IFN-α resistance mechanisms of HCV in FFA-treated culture. Since the concentration of commercially available IFN-α and IFN-λ is expressed in different units, we calibrated the concentrations of each IFN (IFN-α or IFN-λ 1) that inhibits 90% HCV replication by measuring Renilla luciferase activity. We found that the IC90 of IFN-α is 100 IU/ml whereas IC90 of IFN-λ is 10ng/ml. We then measured the relative antiviral activity of IFN-α and IFN-λ in the FFA-treated HCV cell culture. Cells were treated with 2.5XIC_90_, 5XIC_90_ or 10X IC_90_ IFN-α or IFN-λ for 72 hours. Antiviral effect was measured by assaying for Renilla luciferase activity in the lysate and expression of core protein in the infected cells by immunostaining. IFN-λ in all concentrations showed a stronger antiviral effect against HCV and reduction in Renilla Luciferase activity as compared to IFN-α (**Fig 2A**). These results were confirmed by the measurement of intracellular HCV core protein expression via immunostaining (**Fig 2B**). Quantitative assessment of HCV core expression in the IFN-α treated group was made by counting positive cells in 10 high-power fields (40X) and the results were compared with IFN-λ treatment groups (**Fig 2C**). These results indicate that IFN-λ inhibits HCV replication in FFA-treated culture strongly as compared to IFN-α. Co-culture of FFA with HCV-infected cells showed significant impairment of IFN-α and IFN-γ antiviral response (**Fig 3**). This observation is consistent with our previous report [24]. We then compared the long-term HCV clearance of IFN-α and IFN-λ using the FFA-treated HCV cell culture for 35 days. HCV inhibits IFN-α antiviral activity at all time points (13, 20, 27 and 35 days), compared to IFN-λ. These results are statistically significant (p< 0.05). The effect of FFA treatment also prevented IFN-α antiviral activity but these results are not statistically significant. Neither HCV nor FFA treated HCV culture blocked IFN λ antiviral activity. (Fig **4A and 4B**). The results of long-term antiviral treatment response of type I, type II and type III IFN against HCV replication were confirmed by measuring intracellular expression of viral core protein by immunostaining (Fig **4C** and 4**D**). These results conclude that IFN-λ 1 and IFN-λ2 showed a strong and sustained antiviral response against FFA-treated HCV cell culture. Neither IFN-α nor IFN-γ cleared HCV replication in the HCV cell cultures treated with or without FFA.

### IFN-λ inhibits HCV replication in FFA-treated culture through the activation of Jak-Stat signaling

**Fig 5:**
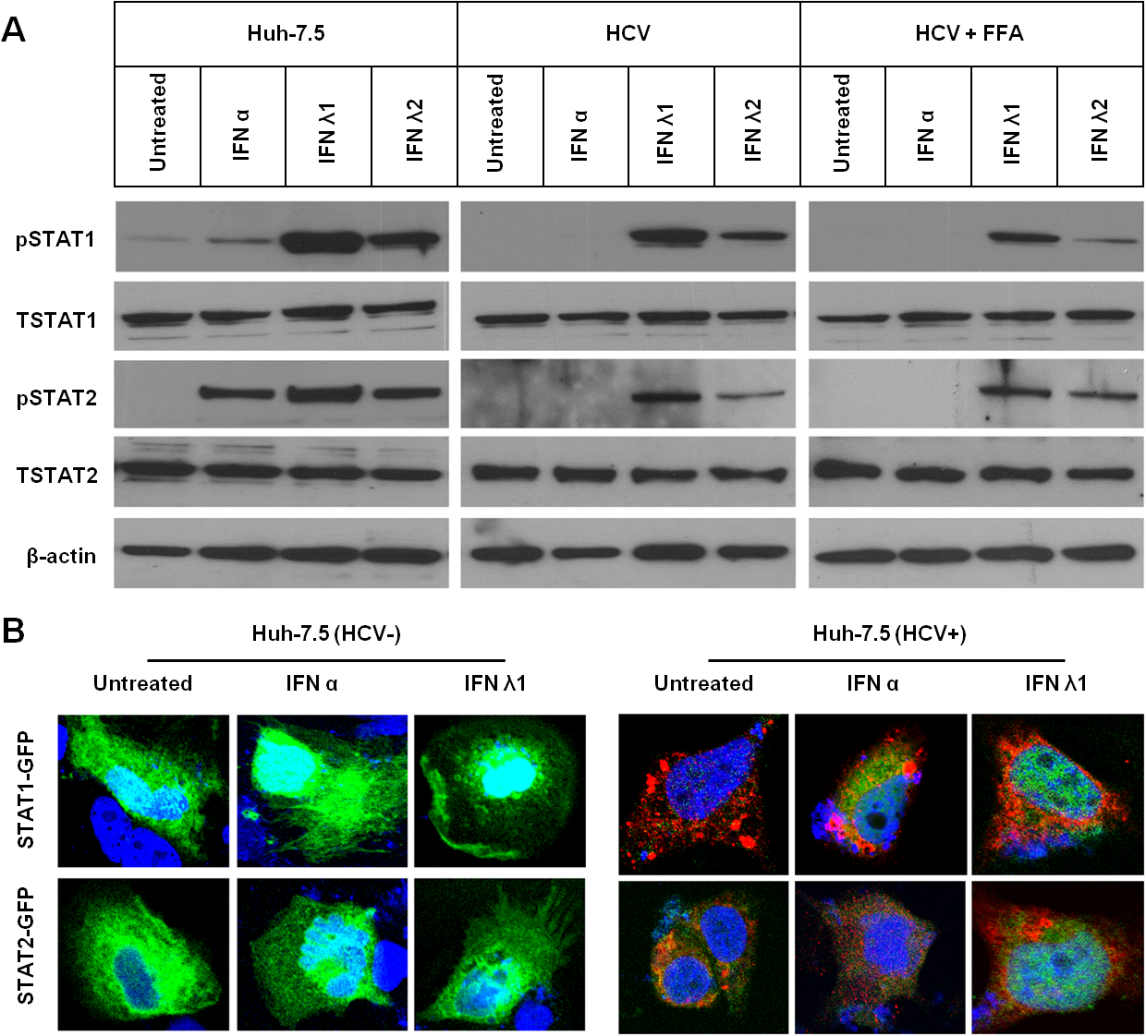
Persistent HCV infected cell culture with or without FFA treatment impairs IFN-α induced phosphorylation and nuclear translocation of Stat1, Stat2. **(A).** Uninfected (HCV-), infected Huh-7.5 cells (HCV+) or infected plus FFA treated cells were treated with IFN-α or IFN-λ or IFN-λ2 for 30 minutes. Equal protein amounts were separated on SDS-PAGE gel and Western blotting was performed using antibodies to p-Stat1, Stat, pStat2 and Stat2 and beta-actin. **(B).** Nuclear translocation of pSTAT1-GFP and pSTAT2-GFP in the uninfected (left panel) and HCV infected culture (right panel) treated with IFN-α or IFN-λ1. HCV core was detected by immunofluorescense. Red represents HCV core and green represents GFP and blue is nuclear staining.

**Fig 6:**
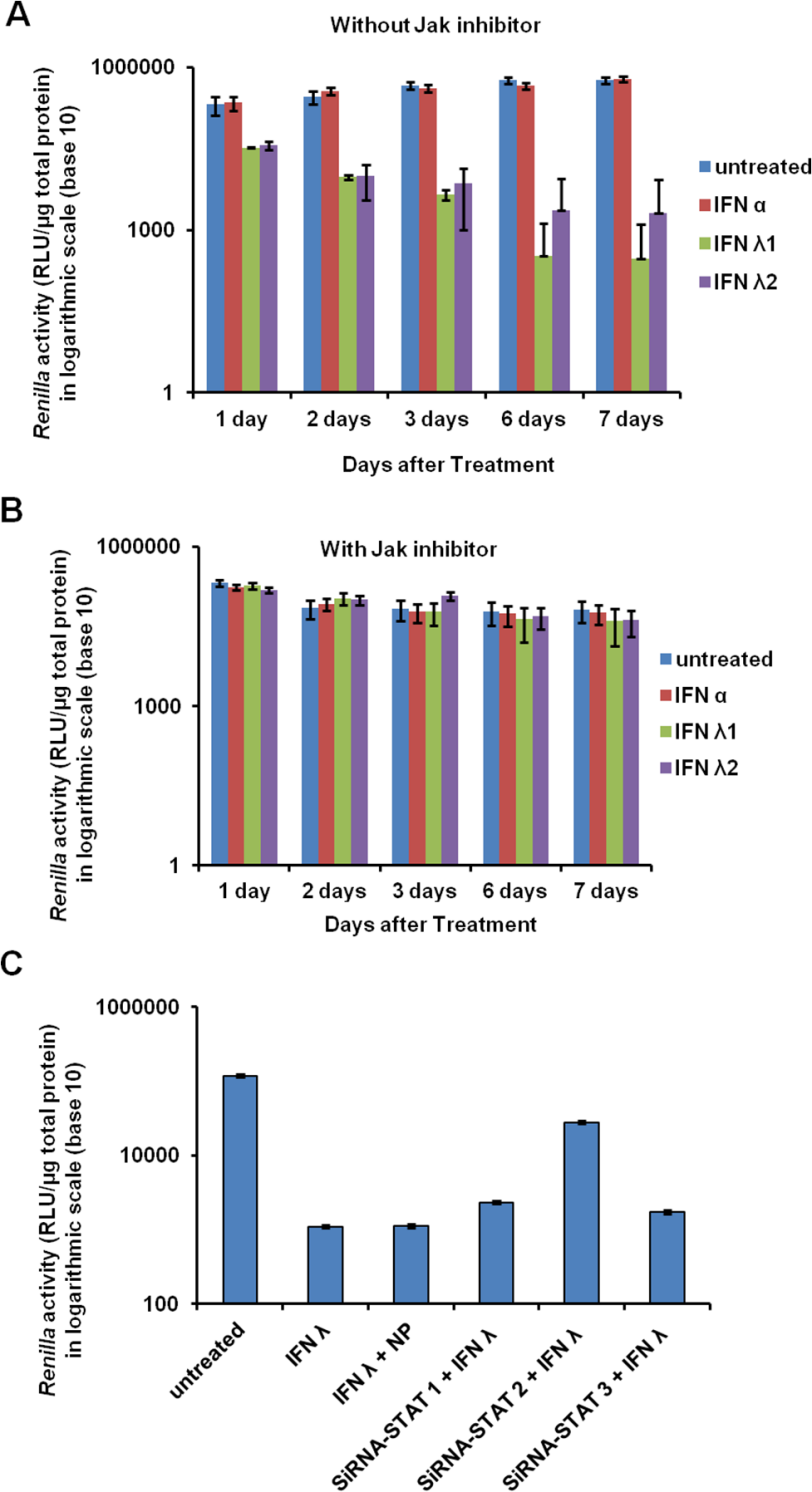
Jak inhibitor and siRNA targeted to Stat2 prevent IFN-λ antiviral mechanisms against HCV in FFA treated cell culture. (**A and B**). FFA treated HCV culture is treated with Jak1 inhibitor for seven days. Antiviral activity of IFN-αand IFN-λwas determined each day by Renilla Luciferase. (**C**). FFA treated HCV cell culture was treated with siRNA targeting Stat1, Stat2 and Stat3 and after 24 hours culture was treated with IFN-λ. After 72 hours antiviral effect of was determined by Luciferase assay. Silencing Stat2 only prevent IFN-λantiviral activity against HCV in FFA treated culture.

The antiviral mechanism of IFN-α and IFN-λ against HCV involves the activation of cellular Jak-Stat signaling. Therefore, Jak-Stat signaling in the FFA-treated HCV cell culture was examined after treatment with comparable amounts of IFN-α or IFN-λ. Whole cell lysates were examined for Stat1 and Stat2 phosphorylation with Western blot analysis. IFN-λ1 and IFN-λ2 induced much stronger activation of Stat1 and Stat2 phosphorylation than cells treated with IFN-α (**Fig 5A**). IFN-λ1 showed stronger activation of Stat1 and Stat2 than IFN-λ2. It is well known that the activated Stat1 and Stat2 proteins enter the nucleus to facilitate IFN-induced antiviral gene transcription. The nuclear translocation of green fluorescence labeled Stat1 and Stat2 in the FFA-treated HCV culture was examined using a confocal microscope. As shown in **Fig 5B**, IFN-α induced nuclear translocation of Stat1 and Stat2 in uninfected Huh-7 cells but prevented it in FFA-treated HCV cell culture, whereas IFN-λ1 induced efficient nuclear translocation of Stat1 and Stat2 protein in FFA-treated HCV culture. To verify that the antiviral response of IFN-λ against HCV is mediated by Jak-Stat signaling, the antiviral activity of IFN-λ against HCV in FFA-treated culture was examined in the presence of siRNA-targeted to Stat1 and Stat2. As shown in **Fig 6A and 6B**, a pan-Janus kinase (JAK) inhibitor Pyridone-6 (Calbiochem, San Diego, CA) used at 1 μM prevented the antiviral effect of IFN-λ1 and IFN-λ2 against HCV in cell culture. The contribution of individual Stat proteins (Stat1, Stat2 and Stat3) in the antiviral mechanisms of IFN-λ1 against HCV in the FFA-treated culture was determined after silencing by siRNA. As shown in **Fig 6C**, Stat2 silencing prevented the antiviral effect of IFN-λ1 and IFN-λ2 suggesting that Stat2 is the most important molecule in the antiviral mechanism.

### FFA-treated HCV cell culture shows impaired expression of cytosolic as well as membrane IFNAR1 receptor

**Fig 7:**
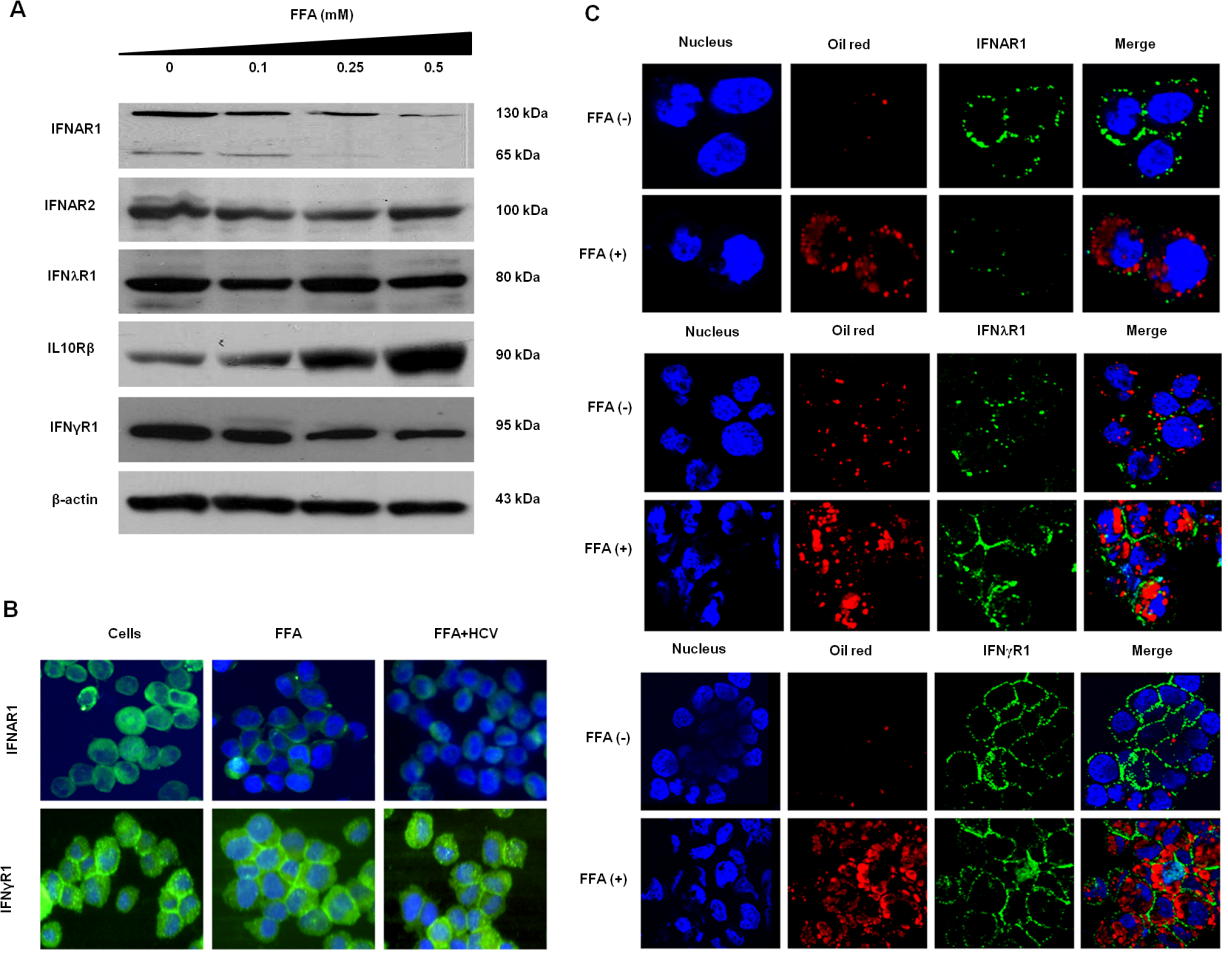
FFA treatment for 48 hours results in down-regulation of cytosolic and membrane expression IFNAR1 in Huh-7.5 cells. **(A).** Western blot showing downregulation of cytosolic low molecular weight IFNAR1 as well as mature high molecular weight IFNAR1 in Huh-7.5 cells treated with FFA in a concentration dependent manner. The expression of IFN-λ1 and IFN-λ receptor was not altered. **(B).** Expression of cytosolic as well as membrane IFNAR1, IFNGR and IFNLRs in FFA treated Huh-7.5 cells. **(C).** The surface expression of IFNAR1, IFNGR and IFNLR1 in FFA treated Huh-7.5 cells by confocal microscopy.

To find an explanation for why HCV replication in FFA-treated culture is sensitive to IFN-λ1 but not to IFN-α, we conducted a systematic analysis of expression levels of two interferon receptors using Western blot analysis, immunofluorescence microscopy and confocal microscopy. Huh-7 cells were treated with different concentrations of FFA, then the expression of IFN-α, IFN-γ and IFN-λ receptors were examined. As shown in **Fig 7A**, FFA treatment downregulated IFNAR1 in a dose-dependent manner, whereas the expression of IFN-α receptor-2, IFN-γ receptor and IFN-λ receptors were not changed. This finding is consistent with our previous study, which confirmed that IFNAR1 expression is decreased in FFA-treated HCV culture. The predicted size of IFNAR1 is 58-60kD, but the high molecular weight band corresponding to molecular weight of 110-130kD represents the actual molecular weight of IFNAR1. This is because there are 15 glycosylation sites present in the IFNAR1 indicating this protein is a highly glycosylated protein [31]. Previously, investigators have shown that treatment of cell extracts with endoglycosylase PNGase F produces low molecular weight IFNAR1 [31]. In the FFA-treated HCV cell culture, we found that expression of both low and high molecular weight IFNAR1 was degraded (**Fig 7A**). IFN-λ receptor levels did not change in FFA-treated HCV cell culture. Interestingly, FFA treatment instead shows a dose-dependent induction of IL-10RB, a receptor used by IFN-λ for its antiviral activity. To verify the significance of the Western blot results, intracellular cytoplasmic expression of IFNAR1 was evaluated with fluorescence microscopy by formaldehyde fixation of FFA-treated HCV-infected cells. These results indicate that FFA-treated HCV cell culture decreases intracellular expression of IFNAR1 (**Fig 7B**). The downregulated expression of cell surface IFN-α receptor-1 was examined by confocal microscopy using a standard protocol that involves staining at 4°C. We found that the cell surface expression of IFNAR1 was impaired in FFA-treated HCV cell culture, but the expression of IFN-γ receptor or IFN-λ receptor remained unchanged (**Fig 7C**). Taken together, these results support our previous findings indicating that the loss of IFNAR1 expression in the FFA-treated culture is the principal reason for the different antiviral responses of IFN-α and IFN-λ against HCV.

### FFA treatment induced autophagy in Huh7.5 cells

**Fig 8:**
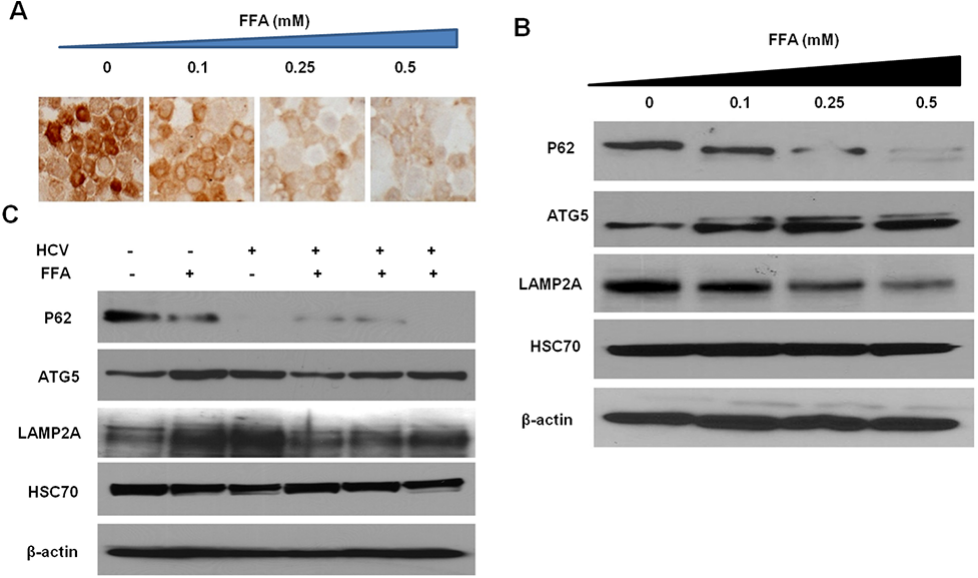
FFA treatment for 48 hours induced autophagy response in Huh-7.5 cells. Huh-7.5 cells were treated with increasing concentrations of FFA and after 72 hours and examined for autophagy induction by immunostaining and Western blot analysis. (**A)** Immunostaining showing expression of p62 in FFA treated Huh-7.5 cells. (B). Western blot show that p62 levels are decreased in FFA treated cells. ATG5 level are induced in FFA treated Huh-7.5 cells. LAMP2A and HSC70 levels and b-actin levels were not altered significantly as compared to untreated Huh-7.5 cells. (C). The expression of p62 was decreased by both HCV and FFA treated culture. The expression of ATG5 and LAMP2A was induced by HCV infected and FFA treated Huh-7.5 cells.

Previously we have shown that HCV infection of Huh-7.5 cells induces ER-stress and autophagy responses leading to the degradation of IFNAR1. We set out to determine whether FFA treatment induces autophagy response, which would explain the increased loss of IFNAR1 in this co-culture model. In a previous study, we have shown that hepatocellular carcinoma cells have insufficient autophagy, and therefore express high levels of p62 proteins [32]. We have shown previously that HCV infection-induced autophagy response selectively degrades p62 in Huh-7.5 cell line [26]. We also found that FFA-induced autophagy response in Huh-7.5 cells clears the expression of p62 (**Fig 8A**). FFA treatment of Huh-7.5 cells induced autophagy, which was confirmed by Western blot analysis of p62, LAMP2A, ATG5 and HSC70 (**Fig 8B**). The autophagy response is induced more when HCV infected cells were cultured with FFA. The levels of LAMP2A induced in HCV cell culture model as well as FFA treatment indicate that the lysosomal biogenesis is also induced (**Fig 8C**).

### Autophagy inhibitor or lysosomal inhibitors prevent IFNAR1 degradation

**Fig 9:**
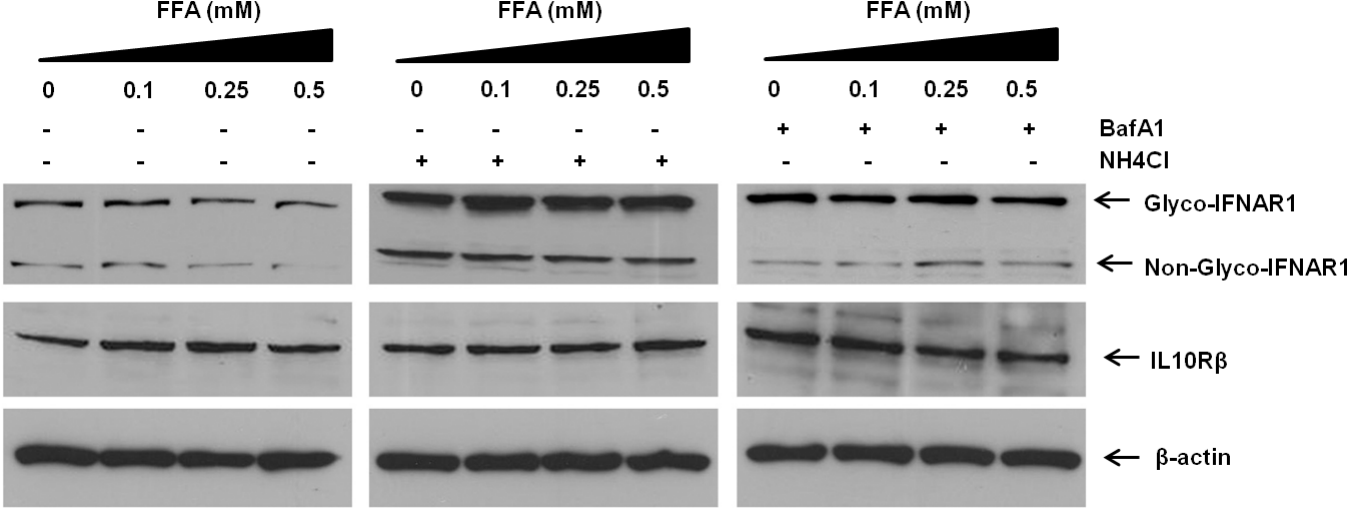
FFA treatment for 48 hours induced decrease of IFNAR1 in Huh-7.5 cells is mediated by a lysosomal degradation pathway. Huh-7.5 cells were treated with FFA for 24 hours in the presence or absence of lysosome inhibitors such as: NH4Cl (30mM) or BafilomycinA1. The expression levels IFNAR1, IL10Rβ and β-actin were examined using the cell lysates by Western blotting.

The inhibition of IFNAR1 in the FFA-treated HCV cell culture model could be explained by three different mechanisms: inhibition of mRNA transcription, inhibition of translation, or protein degradation. We could not find any difference in the IFNAR1 mRNA levels between uninfected and HCV-infected Huh-7.5 cells; rather, the mRNA levels of IFNAR1 are induced after HCV infection (data not shown). Previously in two separate studies we showed that inhibition of ER-stress or autophagy gene restores the expression of IFNAR1, which indicates that the loss of IFNAR1 is at the level of protein degradation [26]. To determine whether the protein degradation pathway due to autophagy induction is responsible for depletion of IFNAR1 in the FFA-treated HCV culture, the IFNAR1 level was examined in FFA-treated cells with ammonium chloride or Bafilomycin A. These results indicate that either the inhibition of lysosomal pH by ammonium chloride or the inhibition of autophagy at the level of autophagolysosome formation by Bafilomycin A1 prevented IFNAR1 degradation (**Fig 9**).

### CMA activation depletes IFNAR1 expression in the FFA-treated HCV cell culture

**Fig 10:**
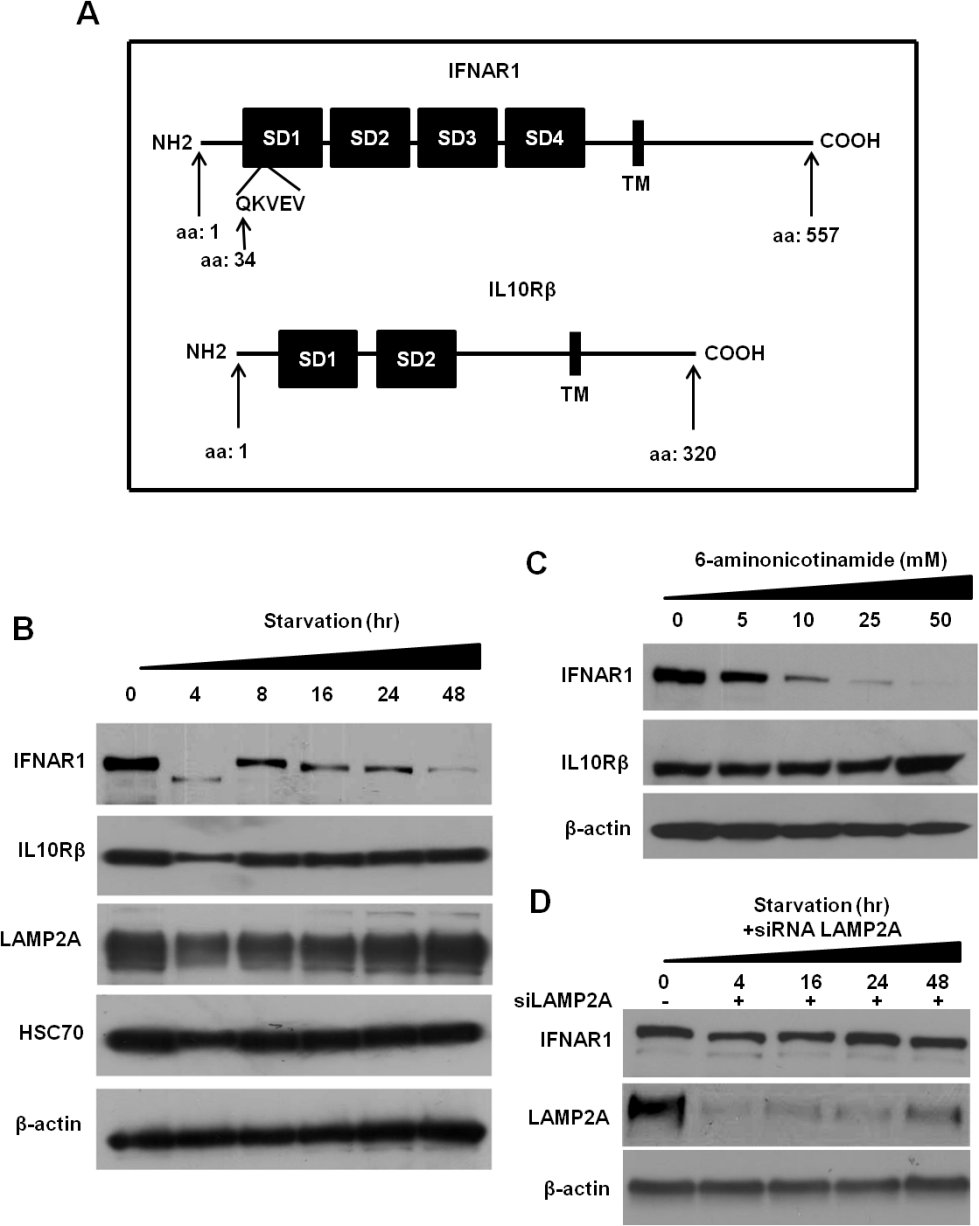
Chaperone induced autophagy targets the degradation of IFNAR1 in HCV and FFA treated Huh-7.5 cells for 48 hours. **(A).** Schematic representation of IFNAR1 protein where presence of CMA motif site is marked. **(B).** Uninfected Huh-7.5 cells were cultured in a serum free medium for indicated time points. The expression of IFNAR1 and IFNLR1 as well as CMA proteins (LAMP2A and HSC70) levels were measured by Western blotting. **(C).** Show the dose-dependent reduced expression of IFNAR1 in the Huh-7.5 cells treated with 6-aminonicotinamide (mM) by Western blotting. The expression of IFN-λ receptor did not change by similar treatment. (**D**). Measurement of IFNAR1 expression after silencing LAMP2A. Huh-7.5 cells were transfected with 60pmole of siRNA using lipofectamine. After 24 hours, cells were serum starved for 4 to 48 hours and cell lysates were measured for IFNAR1 expression by Western blot analysis.

Since lysosome-associated membrane protein type 2A (LAMP2A) levels are induced in FFA-treated HCV cell culture, we investigated whether selective degradation of IFNAR1 could involve chaperone-mediated autophagy (CMA). CMA is a type of autophagy responsible for selective degradation of cytosolic protein bearing certain consensus amino acid motif (KFERQ); the targeting sequence is found to be glutamine (Q) flanked at either end by a hydrophobic (F, I, L, V), an acidic (E, D), a basic (R, K), and the second hydrophobic or basic amino acid [33,34]. Any cytosolic protein containing this motif is recognized by HSC70, a cytosolic chaperone protein called the heat-shock cognate protein. This protein complex then translocates to the lysosome surface where the complex binds to CMA receptor LAMP2A [35]. The protein complex then translocates to the lysosome where the CMA substrates are rapidly degraded. We found that there is a CMA consensus amino acid motif (QKVEV) present at amino acid 34 in the human IFNAR1 protein and this motif is absent in the IFN-λ receptor (**Fig 10A**). The established experimental model used by a number of investigators to activate CMA in cell culture includes either serum starvation or treatment with a small molecule drug called 6-mainonicotinamide (6-AN) [35,36]. Uninfected Huh-7.5 cells with or without FFA treatment were cultured in serum free media for 0, 4, 16 and 48 hours and the expression of IFNAR1 and IFN-λ receptor was examined by Western blot analysis (**Fig 10B**). We found that IFNAR1 expression is significantly diminished after 4 hours of serum starvation and this has no effect on the expression of IFN-λ receptor. Serum starvation also induced the expression of LAMP2A and HSC70 levels. The expression of IFNAR1 levels was decreased in a concentration dependent manner in Huh-7.5 cells treated with 6-aminonicotinamide (6-AN) by Western blotting (**Fig 10C**). Next step, we tested whether silencing LAMP2A by siRNA could rescue the IFNAR1 expression under the serum starvation condition. Huh-7.5 cells were transfected with 60 pmole of siRNA targeted to LAMP2A by using Lipofectamine. After 24 hours, culture media was replaced with serum free media and cells were collected at different time point for analysis. Western blot analysis was performed to verify the effect of LAMP2A silencing of IFNAR1 expression (**Fig 10D**). These results indicate that silencing of LAMP2A in Huh-7.5 cells prevented IFNAR1 degradation due to the serum starvation.

### IFNAR1 interacts with key CMA effectors

**Fig 11:**
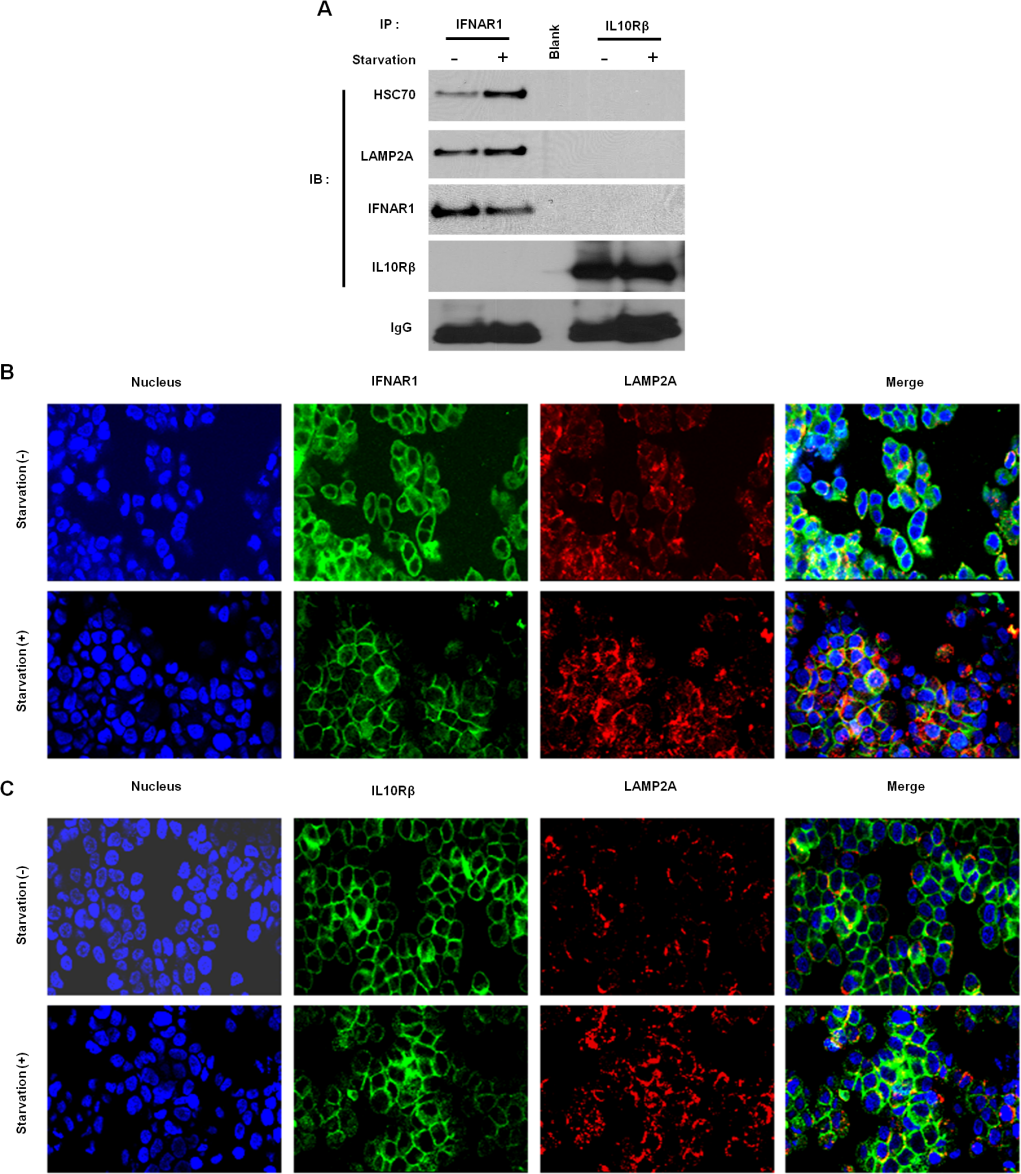
IFNAR1 binds to LAMP2A and localizes to CMA-associated lysosomes. (**A**). Co-immunoprecipitation assay of Huh-7.5 cells serum starved for six hours, after which cells were lysed, and lysates were immunoprecipitated with either IFN-λ receptor or IFN-α receptor and probed for LAMP2A or HSC70 by Western blot analysis. (**B**). Effect of CMA on IFNAR1 localization with LAMP2A. Confocal images of cellular localization of IFNAR1 (green) and LAMP2A (red) in Huh-7.5 cells cultured with serum free medium. DAPI was used for nuclear stain. Only serum starvation enhances CMA, which is associated with increase signal co-localization (red+green = yellow) indicating that IFNAR1 localizing to CMA associated lysosomes for degradation. (**C**) Effect of CMA on IFN-λ receptor localization with LAMP2A. There was no increase in signal co-localization.

Uninfected Huh-7.5 cells serum starved for CMA requires the activity of the cytosolic chaperone HSC70, which binds to the target proteins and mediates their interaction with LAMP2A. This complex then translocates to the lysosome for degradation. Since the lysosome-associated membrane protein type 2A serves as a receptor for the selective uptake and degradation of IFNAR1-HSC70 complex, we verified their interaction with co-immunoprecipitation experiments. Huh-7.5 cells were cultured in serum free media for 4 hours then subjected to immunoprecipitation using antibodies against either IFNAR1 or IFN-λ receptor. Co-immunoprecipitation assay demonstrated an interaction between IFNAR1, and LAMP2A (**Fig 11A**). We have consistently observed that the expression of IFNAR1 decreased upon serum starvation. Co-localization of LAMP2A and IFNAR1 show that CMA active serum starved Huh-7.5 cells showed both the proteins located on the lysosomal membrane (**Fig 11B**). The expression of IFN-λ receptor expression was not altered. Interaction between the LAMP2A, and IFNAR1 on the lysosome membrane was verified by confocal microscopy (**Fig 11C**). These results now support our hypothesis regarding the activation of CMA pathway in FFA-treated HCV cell culture and selective degradation of IFNAR1.

## Discussion

Using an in vitro cell culture system, we show that lipid droplet accumulation in HCV-infected Huh-7.5 cells is significantly higher than in uninfected cells, which supports the clinical findings explaining the higher prevalence of hepatic steatosis in chronic HCV infection compared to the general population. These results are in agreement with findings of other researchers indicating that HCV core protein can induce hepatic steatosis in cell cultures as well as in transgenic mice [37,38]. The level of HCV replication is increased in the hepatocytes supplemented with free fatty acids, which may be why chronic HCV patients with fatty liver show progressive liver damage and hepatic steatosis. Hepatic steatosis, a frequent histological feature of chronic HCV infection, has been reported to be associated with poorer response to antiviral therapy in two large cohort studies [17, 39]. The mechanism by which hepatic steatosis in HCV infection impairs IFN-α antiviral response is not clear. We took advantage of an infectious HCV cell culture model to investigate how hepatocellular steatosis affects HCV replication and antiviral response. Our results show that co-culture of HCV with free fatty acids induced macrovesicular steatosis, which prevented IFN-α antiviral activity by downregulating the expression of IFN-α receptor-1. Treatment of uninfected Huh-7.5 cells with a mixture of oleate and palmitate (FFA) induced cellular autophagy response and downregulated the expression of IFNAR1. Downregulation of IFNAR1 leads to impaired phosphorylation, nuclear translocation and IFN-α antiviral response in FFA-treated HCV cell culture model. In this study, we showed that FFA-treated HCV culture remained more resistant to IFN-α treatment because of diminished expression of IFNAR1. These results support our previous observation indicating that HCV induced ER-stress and autophagy response impairs IFN-α antiviral response by downregulating the expression of IFNAR1 [26]. A recent study in our laboratory demonstrated that co-culture of alcohol or free fatty acids along with HCV-infected Huh-7.5 cells additively decreased cell surface expression of IFNAR1 [40]. Reduced expression of IFNAR1 has also been observed in the liver biopsies of chronic liver disease patients with hepatitis C virus infection [40]. Therefore, we speculate that any exogenous agent that induces ER-stress and autophagy response additively inhibits the expression of IFNAR1, leading to impairment of IFN-α treatment response against HCV. Chronic ER-stress and autophagy response also affect RBV antiviral activity by downregulating membrane expression of ENT1 [41]. Our results provide a mechanism for how FFA-treated HCV cell culture impairs the antiviral response of IFN-α.

Multiple HCV genotypes have been isolated worldwide. Genotype appears to be involved in the main pathological aspects of HCV infection. Insulin resistance, steatosis and progression toward cirrhosis, fibrosis and hepatocellular carcinoma establish and develop following genotype-specific mechanisms. Moreover genotype influences pharmacological treatment in term of dose and duration. Pathways involved in cell proliferation, apoptosis, lipid metabolism, insulin and interferon signaling are impaired to a different extent among genotypes, leading to distinct pathological settings. Genotype 1 is associated with a more aggressive disease with increased insulin resistance, worst response to therapy, higher risk of cirrhosis and hepatocellular carcinoma development, while genotype 3 is associated with increased steatosis and fibrosis. The identification and characterization of HCV types and subtypes provides insight into the different outcomes of HCV infection and responsiveness to therapy. In the present article, all these experiments were performed with JFH1 clone, which is based on HCV genotype 2a backbone. A report by Abid et al. indicates that highest level triglyceride accumulation in Huh-7 cells transfected HCV genotype 3a core protein compared to HCV genotype 1b and 2a, indicating that lipid accumulation is strongly affected by HCV genotype [42]. The impact of HCV genotypes on the pathobiology of liver disease were reviewed by Ripoli et al indicating that genotype 3 is associated with increased steatosis and fibrosis [43]. Therefore, future studies should be performed to verify whether HCV genotypes 3 culture that favors lipid accumulation could also prevent IFN-α treatment response.

The cellular autophagy mechanism does not alter the expression of IFN-λ receptor, which is a type III IFN receptor. We show that IFN-λ induces phosphorylation and nuclear translocation of Stat proteins and activates the same Jak-Stat signaling to induce HCV clearance. We found that IFN-λ activates Stat1 and Stat 2 phosphorylation more strongly than IFN-α in FFA-treated cell culture model. Our results are consistent with many others who have found that IFN-λ activates Jak-Stat signaling for its antiviral clearance [44,45]. This study also provides some insight into the role of IFN-λ and HCV clearance geared through many clinical studies linking the IL-28B genetic polymorphisms with HCV treatment clearance. At present, the mechanism by which the IL-28B genotype affects HCV clearance has not been well established; one possibility is that IL-28B genetic information relates to the production of IFN-λ level during chronic HCV infection.

The second part of this study provides a potential mechanism for how IFNAR1 expression is downregulated in HCV and FFA-treated HCV cell culture model. FFA-treated HCV culture showed reduced expression of IFNAR1 and was unable to induce Stat phosphorylation or nuclear translocation upon IFN-α treatment. We also investigated the selective degradation of IFNAR1 in HCV and FFA-treated cell culture. Two different mechanisms have been proposed to explain why IFNAR1 protein is degraded in viral infection. One possibility is that virus-induced ER-stress response (UPR/ER stress) can downregulate the expression of IFNAR1 [46] Another mechanism that explains the reduced expression of IFNAR1 relates to the ligand-induced degradation of IFNAR1 through autophosphorylation at S535 and S539, which creates a target site for ubiquitination and degradation [47,48]. In addition to these two mechanisms, our study provides another novel autophagy-related mechanism that selectively degrades IFNAR1. Cellular autophagy can be classified into three major types: macroautophagy, chaperone-mediated autophagy (CMA) and microautophagy. To find an explanation why HCV selectively degrades IFNAR1, and spares type III IFN receptors, we investigated the role of CMA. CMA is a process where cytosolic proteins are removed selectively from the cytoplasm and delivered to the lysosome surface for degradation. CMA can be activated by serum starvation or nutrient starvation. Our results show that IFNAR1 is degraded by serum starvation and the expression of IFN-λ receptor is not affected. Substrate for CMA includes an amino acid motif (KFERQ) present in IFNAR1 but on the IFN-λ receptor. This provides an explanation for why HCV-induced cellular autophagy response specifically targets IFNAR1 but not the IFN-λ receptor. We showed that HCV and FFA-induced autophagy response induces LAMP2A protein expression. Activated CMA allows specific interaction between HSC70, LAMP2A and IFNAR1, as supported by our results. Taken together, we believe that IFNAR1 degradation occurs in the lysosome by CMA. Many RNA and DNA viruses develop persistent infection of human cells by overcoming cellular innate and adaptive immunity. The CMA-induced degradation of IFNAR1 by an FFA-treated HCV culture model provides a potential explanation for HCV’s ability to overcome innate and adaptive immunity, and why humans frequently develop chronic HCV infection.
