# Expression of Concern: Chaperone-Mediated Autophagy Targets IFNAR1 for Lysosomal Degradation in Free Fatty Acid Treated HCV Cell Culture

**DOI:** 10.1371/journal.pone.0319262

**Published:** 2025-02-07

**Authors:** The PLOS One Editors

After publication of this article [1], concerns were raised about results presented in Figs 1 and 4. Specifically:

- The Fig 1A HCV(-) panel appears similar to the Fig 1D (0) panel.
- The upper part of the Fig 4B 20d Untreated panel appears similar to the lower part of the Fig 4D 20d Untreated panel, for which the reported experimental conditions are different.

The corresponding author stated that the primary data underlying this article [1] are no longer available, and the primary data were not provided with the published article [1], contrary to the Data Availability statement. This article [1] therefore does not comply with the PLOS Data Availability Policy.

In light of the concerns listed above that have not been resolved, and as this article [1] does not comply with the PLOS Data Availability Policy, the *PLOS One* Editors issue this Expression of Concern.

## References

[1] Kurt R, Chandra PK, Aboulnasr F, Panigrahi R, Ferraris P, Aydin Y, et al. (2015) Chaperone-Mediated Autophagy Targets IFNAR1 for Lysosomal Degradation in Free Fatty Acid Treated HCV Cell Culture. PLoS ONE 10(5): e0125962. 10.1371/journal.pone.0125962 25961570 PMC4427131

